# Sensitive Indirect Spectrophotometric Method for Determination of H_2_-Receptor Antagonists in Pharmaceutical Formulations

**Published:** 2007-06

**Authors:** Ibrahim A. Darwish, Samiha A. Hussein, Ashraf M. Mahmoud, Ahmed I. Hassan

**Affiliations:** 1 *Department of Pharmaceutical Analytical Chemistry, Faculty of Pharmacy, Assiut University, Assiut 71526, Egypt;*; 2 *Department of Pharmaceutical Analytical Chemistry, Faculty of Pharmacy, Al-Azhar University, Assiut, 71524, Egypt*

**Keywords:** H_2_-receptors antagonists, cerium (IV), p-dimethylaminocinnamaldehyde, spectrophotometry, pharmaceutical analysis

## Abstract

A simple, accurate and sensitive spectrophotometric method has been developed and validated for determination of H_2_-receptor antagonists: cimetidine, famotidine, nizatidine, and ranitidine hydrochloride. The method was based on the oxidation of these drugs with cerium (IV) in presence of perchloric acid and subsequent measurement of the excess Ce (IV) by its reaction with p-dimethylaminocinnamaldehyde to give a red colored product (λ_max_ at 464 nm). The decrease in the absorption intensity (ΔA) of the colored product, due to the presence of the drug was correlated with its concentration in the sample solution. Different variables affecting the reaction were carefully studied and optimized. Under the optimum conditions, linear relationships with good correlation coefficients (0.9985-0.9994) were found between ΔA values and the concentrations of the drugs in a concentration range of 1-16 µg ml^-1^. The assay limits of detection and quantitation were 0.12-0.44 and 0.37-1.33 µg ml^-1^, respectively. The method was validated, in terms of accuracy, precision, ruggedness, and robustness; the results were satisfactory. The proposed method was successfully applied to the analysis of the investigated drugs in their pure and pharmaceutical dosage forms (recovery was 98.8-102.5 ± 0.79-1.72%) without interference from the common excipients. The results obtained by the proposed method were comparable with those obtained by the official methods.

## INTRODUCTION

**Figure 1:**
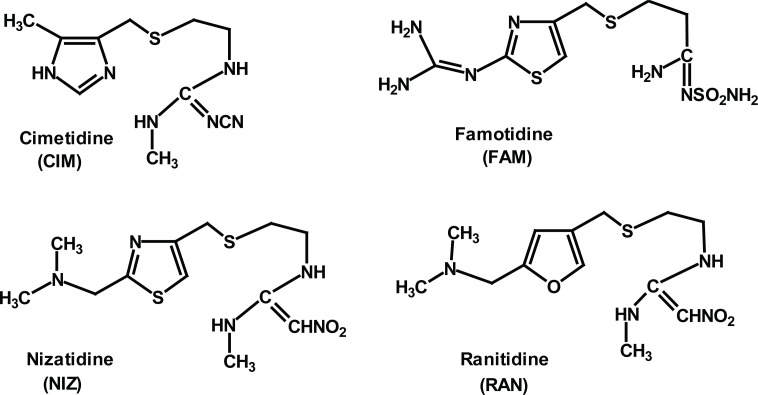
The chemical structure of the investigated H_2_-receptor antagonists.

Histamine H_2_-receptor antagonists (H_2_-RAs), competitively inhibit the action of histamine on the histaminic H_2_-receptors of parietal cells, and thus reduce the gastric acid secretion. Therefore, these drugs are used for treatment of active duodenal ulcer, active and benign gastric ulcer, pathogenic gastrointestinal hypersecretory conditions (e.g. Zollinger-Ellison Syndrome), and symptomatic relief of gastroesophageal refluxes (1-3). Four H_2_-RAs are presently available and extensively used in our community. These are cimetidine (CIM), famotidine (FAM), nizatidine (NIZ), and ranitidine (RAN); their chemical structures are given in Figure 1.

Because of the clinical success and wide use of H_2_-RAs, several methods have been reported for their determination in bulk, and pharmaceutical dosage forms. These methods include titrimetry (4-6), electrochemical methods (7), TLC (8), HPLC (9), capillary electrophoresis (10), and fluorimetry (11, 12). Spectrophotometry is considered more convenient alternative technique because of its inherent simplicity, adequate sensitivity, and availability in all quality control laboratories. Unfortunately, the spectrophotometric methods reported for determination of H_2_-RAs (13-18) were associated with some drawbacks as lack of sensitivity, time-consuming, laborious multiple procedures, and/or require critical expensive derivatizing reagents. The present study describes the development of new simple spectrophotometric method that overcomes these drawbacks.

The analytical procedure of the present work involved the oxidation of the H_2_-RAs with excess Ce (IV) and subsequent measurement of the remaining unreacted Ce (IV) by its reaction with p-dimethylaminocinnamaldehyde (DMAC) to give red colored product that was measured at 464 nm.

## EXPERIMENTAL

### Apparatus

UV-1601 PC (Shimadzu, Kyoto, Japan) and Lambda-3 B (Perkin-Elmer Corporation, Norwalk, USA) ultraviolet-visible spectrophotometers with matched 1-cm quartz cells were used for all measurements.

### Materials and reagent solutions

Cimetidine (CIM; Sigma Chemical Co., St. Louis, MO, USA), famotidine (FAM; Sigma Chemical Co., St. Louis, MO, USA), nizatidine (NIZ; Eli Lilly Co, Indianapolis, IN, USA), and ranitidine HCl (RAN; Glaxo-Wellcome, London, UK) were obtained and used as received. The stock standard solutions (0.8 mg ml^-1^) were prepared by dissolving an accurately weighed amount (40 mg) of the drug in 50 ml water, in a 50-ml calibrated flask. The working standard solutions were obtained by further dilution of this stock solution with water. Ceric ammonium sulphate (Sigma Aldrich Co. Ltd., Gillingham-Dorset, Germany), 0.15% (w/v in 4 M perchloric acid) prepared fresh daily. p-Dimethylaminocinnamaldehyde (DMAC, Winlab Co., UK), 0.02% (w/v) aqueous solution prepared fresh daily. All solvents, acids, and other chemicals used throughout this study were of analytical grade. Double distilled water was obtained through Nanopure II water purification system (Barnstead-Thermolyne, Dubuque, IA, USA), and used throughout the work.

### Pharmaceutical formulations

Famotin^®^ tablets (Memphis, Cairo, Egypt), Antodine^®^ tablets (Amoun Pharmaceutical Industries, Cairo, Egypt), Servipep^®^ tablets (Novartis Pharma, Cairo, Egypt), Peptic^®^ tablets (Julphar, U.A.E), Famotak^®^ tablets (South Egypt Industries Company, Cairo, Egypt), Gastrodomina^®^ tablets (Medical Union Pharmaceuticals, Ismailia, Egypt), and Antodine^®^ ampoules (Amoun Pharmaceutical Industries, Cairo, Egypt) are labeled to contain 40 mg of FAM per tablet or ampoule. Nizatin^®^ capsules (Hi Pharm, Cairo, Egypt) are labeled to contain 300 mg of NIZ per capsule. Ranitidol^®^ tablets (El-Nasr Pharmaceutical Chemicals, Cairo, Egypt) are labeled to contain 150 mg of RAN per tablet. Ranitak^®^ tablets (South Egypt Industries Company, Cairo, Egypt) are labeled to contain 300 mg of RAN per tablet. Zantac^®^ tablets (Glaxo-Welcome Egypt S.A.E., El-Salaam City, Cairo, Egypt), and Ranitidine^®^ tablets (Medical Union Pharmaceuticals, Ismailia, Egypt), Aciloc^®^ tablets (Sigma, Cairo, Egypt are labeled to contain 300 mg of RAN per tablet. Zantac^®^ ampoule (Glaxo-Welcome Egypt S.A.E., El-Salaam City, Cairo, Egypt), and Ranitidine^®^ ampoule (Medical Union Pharmaceuticals, Ismailia, Egypt) are labeled to contain 50 mg of RAN per ampoule. Cimetidine tablets simulated in the laboratory according to reported formulation labeled to contain 300 mg of CIM per tablet.

### Preparation of pharmaceutical dosage form samples

**Tablets and capsules.** Twenty tablets or the contents of 20 capsules were weighed, and finely powdered. An accurately weighed quantity of the powdered tablet or capsule contents equivalent to 200 mg of the active ingredient was transferred into a 100 ml calibrated flask, and dissolved in about 50 ml of water. The contents of the flask were swirled, sonicated for 5 min, and then completed to the volume with water. The mixtures were mixed well, filtered and the first portion of the filtrate was rejected. A measured volume (5.0 ml for CIM and NIZ, and 1.5 ml for FAM and RAN) of the prepared solution was diluted quantitatively to 100 ml with the distilled water, and the resulting solution was used for analysis by the recommended procedure.

**Ampoules.** The contents of five ampoules were quantitatively transferred into a 250 ml calibrated flask and completed to the mark with water. A measured volume (1.5 ml) of the prepared solution was diluted quantitatively to 50 ml with the distilled water, and the resulting solution was used for analysis by the recommended procedure.

### General recommended procedure

One milliliter, accurately measured, of the standard (20-160, 10-70, 20-140, and 10-40 µg ml^-1^ for CIM, FAM, NIZ, and RAN, respectively) or sample solution was transferred into a 10-ml volumetric flask. One milliliter of cerric ammonium sulfate (0.15% w/v in 4 M perchloric acid) was added, mixed well, and allowed to stand, at room temperature (25 ± 5°C), for 15, 20, 20, and 30 min for CIM, FAM, NIZ, and RAN, respectively. Then, 1 ml of 0.02% (w/v) DMAC reagent solution was added, and allowed to stand for another 1 minute. The solution was completed to the mark with distilled water. The decrease in the absorbance (ΔA) was measured at 464 nm against blank treated similarly. Calibration graphs were constructed by plotting the obtained ΔA values versus the corresponding drug concentrations, and the amount of drug in each particular sample was calculated from its corresponding calibration curve.

### Determination of molar ratio of the reactions

**For H_2_-RA drugs with Ce (IV)**. The mole ratio method was employed. One-milliliter aliquots of the drug solution (4.5 × 10^-3^ M) were transferred into 25 ml calibrated flasks. To each flask, 1-20 ml aliquots of Ce (IV) solution (4.5 × 10^-3^ M) were added, and the reactions were allowed to proceed at room temperature (25 ± 5°C), for 15, 20, 20, and 30 min for CIM, FAM, NIZ, and RAN, respectively. One milliliter of DMAC solution (0.02%, w/v) was added to each flask, and the reaction mixtures were completed to volume with water. The decrease in absorbance was measured at 464 nm against reagent blanks treated similarly, except the drugs were omitted.

**For Ce (IV) with DMAC**. The continuous variation method (19) was also employed. One-milliliter aliquots of Ce (IV) solution (2 × 10^-3^ M) were transferred into 10-ml calibrated flasks. To each flask, 1-10 ml aliquots of DMAC solution (2 × 10^-3^ M) were added. The reactions were allowed to proceed at room temperature (25 ± 5°C) for 15, 20, 20, and 30 min for CIM, FAM, NIZ, and RAN, respectively. The reaction mixtures were completed to volume with water, and the absorbance was measured at 464 nm against reagent blanks prepared without Ce (IV).

## RESULTS AND DISCUSSION

Oxidation-reduction reactions have been used as the basis for the development of simple and sensitive spectrophotometric methods for the determination of many pharmaceutical compounds (20-23). Cerric, Ce (IV), because of its high oxidation potential and excellent solution stability, has been widely used as an effective analytical reagent in these methods (20). In general, the use of Ce (IV) as an analytical reagent is based on either the decrease in its yellow color as a result of the analyte oxidation (21, 22), or measuring the excess reagent by color-developing second reagent (23). The first approach, due to the measuring in UV region, is liable to interferences by UV-absorbing interfering substances. In the second approach, the measurements are performed in the visible region, thus the potential interferences are avoided. Therefore, this approach was considered in the present study.

### Design and strategy for assay development

**Figure 2:**
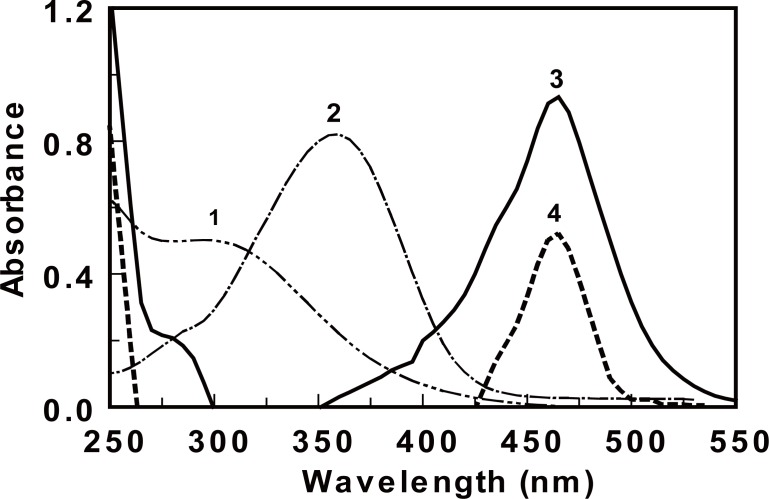
Absorption spectra of Ce (IV) (0.15%, w/v, 1), and DMAC (0.02%, w/v, 2), and the reaction product between Ce (IV) and DMAC in the absence and presence of cimetidine (10 µg ml^-1^, 3 and 4, respectively).

**Reaction involved and absorption spectra.** The proposed procedures involved two steps; the first one was concerned with the treatment of the investigated H_2_-RAs with known excess amount of Ce (IV). The second step involved the determination of the excess unreacted Ce (IV) via its reaction with DMAC reagent. The addition of DMAC to Ce (IV) resulted in the formation of a red colored product that can be measured at 464 nm. The investigated H_2_-RAs and DMAC reagent had no absorption capability at the measuring wavelength. The decrease in the absorption intensity (ΔA) at 464 nm, caused by the presence of the drug, was directly proportional to the amount of the drug in its original sample. Figure 2 illustrates the absorption spectra of the reaction of Ce (IV) with DMAC in presence and absence of cimetidine, as a representative example of H_2_-RAs. Similar pattern of results were obtained with the other H_2_-RAs.

### Optimization of reaction variables

According to the above-mentioned reaction, Ce (IV) should be added in excess to react with the drug substance. By measuring the excess Ce (IV) reagent, the consumed reagent would correspond to the amount of the drug. The highest concentration of Ce (IV) reagent that reacts with a definite concentration of DMAC reagent and gives the highest absorption value within the practical sensitivity range of absorption values (≈0.9) was considered as optimum. These concentrations were found to be 0.15 and 0.02 % w/v (studied range: 0.05-3 and 0.005-0.2% w/v) for Ce (IV) and DMAC, respectively.

The oxidation reaction was conducted in acid medium to avoid the precipitation of hydrated ceric oxide, CeO_2_ × H_2_O. In order to determine the most suitable acid for the reaction, different acids (sulphuric, hydrochloric, nitric, perchloric and acetic) were tested. The results revealed that the reaction of H_2_-RA drugs with Ce (IV) proceeded quantitatively only in the presence of perchloric acid, as the highest ΔA values were obtained. This was attributed to the highest oxidation potential of Ce (IV) in perchloric acid (E_o_=1.75 V) as compared to that of Ce (IV) in 4 M sulfuric (E_o_=1.44 V), nitric (E_o_=1.61 V), hydrochloric (E_o_=1.28 V) or acetic acids (E_o_<1.0 V). Moreover, the oxidation of organic compounds by Ce (IV) in 4 M sulfuric acid was proved to be extremely slow and failed to be stoichiometric. The greater oxidation potential of Ce (IV) in perchloric acid overcomes both the slowness of the oxidation process and the inexact stoichiometry encountered in sulfuric acid. The solutions of Ce (IV) in hydrochloric acid are unstable owing to the oxidation of chloride ions to chlorine gas (24). For these reasons, perchloric acid was selected for the next experiments. The effect of perchloric acid concentration on the reaction was studied using different acid concentrations in the range of 0.5-9 M. The results showed that the ΔA increased by increasing the acid concentration up to 3 M and remained constant until 5 M, then decreased upon using higher concentrations. Therefore, 4 M perchloric acid solution was used for the subsequent experiments.

Under these conditions, the reaction between the investigated H_2_-RAs and Ce (IV) was completed at room temperature (25 ± 5°C) within 15, 20, 20, and 30 min for CIM, FAM, NIZ, and RAN, respectively. The effect of heating temperature on the oxidation of the investigated drugs with Ce (IV) was studied by performing the reaction at room temperature and at elevated temperature ranging from 40-100°C by heating the reactions in a water bath (MLW type thermostatically controlled water bath (Memmert GmbH, Co. Schwa batch, Germany) for different times. The results revealed that the elevated temperatures had no significant accelerating effect on the reaction time. The reaction of DMAC with Ce (IV) was instantaneous as complete color development was achieved immediately after its addition to Ce (IV) solution.

In order to select the proper solvent for dilution, different solvents were tested. The highest ΔA values were obtained when water was used as a diluting solvent. Substitution of water by other solvents (methanol, ethanol, acetone, propanol, and 1, 4-dioxane) resulted in slight bathochromic shifts, and the ΔA values were also decreased. After dilution with water, the ΔA values were found to be stable for at least 30 min.

### Validation of the proposed method

**Linearity, limits of detection and quantitation**. Under the optimum conditions, the calibration graphs correlating the decrease in the absorption intensity (ΔA) with the corresponding concentration of the drug were constructed. Regression analysis for the results were as carried out using least-square method. In all cases, Beer's law plots (n=5) were linear with very small intercepts (-0.0035-0.0078) and good correlation coefficients (0.9985-0.9994) in the general concentration ranges of 1-16 µg ml^-1^ (Table 1). The limits of detection (LOD) and limits of quantitation (LOQ) were determined using the formula: LOD or LOQ=κSD_a_/b, where κ=3 for LOD and 10 for LOQ, SD_a_ is the standard deviation of the intercept, and b is the slope. The LOD and LOQ values ranged from 0.12-0.44 and 0.37-1.33 µg ml^-1^, respectively.

**Table 1:** Quantitative parameters for the spectrophotometric analysis of H_2_-receptor antagonists by the proposed method

Compound	Range (µg ml^-1^)	Intercept (a)	Slope (b)	Correlation coefficient (r)	Molar absorptivity (l mol^-1^cm^-1^)	LOD (µg ml^-1^)	LOQ (µg ml^-1^)

Cimetidine	2.0-16	0.0036	0.0527	0.9989	13660	0.44	1.33
Famotidine	1.0-7	-0.0035	0.1280	0.9986	41780	0.27	0.82
Nizatidine	2-14	0.0078	0.0595	0.9994	19920	0.40	1.23
Ranitidine HCl	1-4	0.0066	0.2070	0.9985	71340	0.12	0.37

**Accuracy and precision**. The accuracy of the proposed method was evaluated by the standard addition method at three different concentrations levels. The recovery values of the added concentrations were 98.7-101.1 ± 0.55-1.15% (Table 2). This indicated the accuracy of the proposed methods. The precisions of the proposed method were determined by replicate analysis of five separate solutions of the working standards at three concentration levels of each drug. The intra-day precision was assessed by analyzing 6 replicates of each sample as a batch in a single assay run, and the inter-day precision was assessed by analyzing the same sample, as triplicate, in two separate runs. The method gave satisfactory results; the relative standard deviations did not exceed 2% indicating the good reproducibility of the proposed method (Table 3). This precision level is adequate for the precision and routine analysis of the investigated drugs in quality control laboratories.

**Table 2:** Results of standard addition method for the proposed spectrophotometric method for determination of H_2_-receptor antagonists

Drug	Amount added (µg µml^-1^)	Recovery (% ± SD)^a^	RSD (%)

Cimetidine (12.5)^b^	12.5	100.7 ± 0.80	0.79
	25	99.6 ± 0.92	0.92
	37.5	99.1 ± 0.73	0.74
Famotidine (5)	5	99.8 ± 1.05	1.05
	10	101.1 ± 0.77	0.76
	15	100.6 ± 0.88	0.87
Nizatidine (10)	10	100.5 ± 1.15	1.14
	20	99.1 ± 0.66	0.67
	30	99.7 ± 0.93	0.93
Ranitidine HCl (2.5)	2.5	99.3 ± 0.55	0.55
	5	99.3 ± 1.11	1.12
	7.5	98.7 ± 0.82	0.83

a Values are the mean of three determinations;

b Figures in parenthesis are the amounts taken in µg.ml^-1^.

**Table 3:** Precision of the proposed spectrophotometric method for determination of H_2_-receptor antagonists

Drug	Conc. (µg ml^-1^)	RSD (%)^a^

Cimetidine	4	1.96
	10	0.83
	16	0.85
Famotidine	2	1.81
	5	0.73
	7	0.47
Nizatidine	4	1.9
	10	0.66
	14	0.57
Ranitidine HCl	1	1.69
	3	0.52
	4	0.50

a Values are mean of three determinations.

**Interference liabilities**. Before proceeding with the analysis of the investigated drugs in their pharmaceutical dosage forms, interference liabilities were carried out to explore the effect of common excipients that might be added during formulations. Samples were prepared by mixing known amount (300 mg) of the drug with various amounts of the common excipients: lactose, sucrose, starch, magnesium stearate, and ascorbic acid (added as stabilizer in the formulation of the ampoule). The analysis of these laboratory-prepared samples was carried out using the general recommended procedure, and the recovery values were determined. No interference was found from lactose, sucrose, starch, talc, gum acacia, glucose, and magnesium stearate; the recovery values were 98.5-102.6 ± 0.62-1.91%. This indicated the absence of interference liabilities from these excipients. Although the method, being based on oxidation reaction is not selective, however, the good recoveries ensured its suitability for the analysis of the investigated drugs in their solid dosage forms without interference from the common reducing excipients. This was attributed to the high sensitivity of the method that necessitated the dilution of the sample, and consequently the excipients beyond their interference capabilities. On the other hand, ascorbic acid, added as a stabilizer, was found to interfere with the assay procedure for analysis of RAN-containing ampoules. This interference could be eliminated by adding 1 ml of 0.1% (w/v) aqueous solution of potassium bromate to the ampoule samples prior to their analysis. Potassium bromate, being mild oxidant, was used in this experiment to oxidize the ascorbic acid; however it was unable to oxidize the drug (RAN). Nevertheless, the proposed method has the advantage that the measurements are performed at 464 nm in the visible region away from the UV-absorbing capabilities of interfering substances that might be co-extracted from dosage forms.

**Robustness and ruggedness**. Robustness was examined by evaluating the influence of small variation of method variables including, concentrations of analytical reagents, and reaction time on the performance of the proposed methods. In these experiments, one parameter was changed whereas the others were kept unchanged, and the recovery percentage was calculated each time. It was found that small changes in these variables did not significantly affect the method; the recovery values were 98.9-100.2 ± 0.60-1.27%. This provided an indication for the reliability of the proposed method during its routine application for analysis of the investigated drugs. Ruggedness was tested by applying the proposed method to the assay of the investigated drugs using the same operational conditions but using two different instruments at two different laboratories and different elapsed time. Results obtained from lab-to-lab and day-to-day variations were found to be reproducible, as RSD did not exceed 2% (Table 4).

**Table 4:** Ruggedness of the proposed spectrophotometric method

Drug	Recovery (% ± SD)^a^				
	Instrument-to-instrument variation		Day-to-day variation		
	Shimadzu	Perkin-Elmer	Day-1	Day-2	Day-3

Cimetidine	100.0 ± 0.77	99.6 ± 0.88	99.9 ± 0.82	100.1 ± 0.65	99.5 ± 0.99
Famotidine	99.3 ± 0.71	98.9 ± 0.70	99.8 ± 1.02	99.5 ± 0.85	99.7 ± 1.11
Nizatidine	99.5 ± 0.98	99.9 ± 0.60	100.2 ± 0.79	99.7 ± 1.05	99.2 ± 0.69
Ranitidine HCl	99.8 ± 0.80	99.3 ± 0.75	99.6 ± 1.13	99.7 ± 1.27	99.5 ± 0.76

a Values are the mean of three determinations ± SD.

### Application of the proposed method to the analysis of dosage forms

It is evident from the aforementioned results that the proposed method gave satisfactory results with the investigated drugs in bulk. Thus their pharmaceutical dosage forms were subjected to the analysis of their contents of the active ingredient by the proposed method and the official method (25). The recovery, as percentages, ranged from 98.8-102.5 ± 0.79-1.72% (Table 5). These results were compared with those obtained from the official method by statistical analysis with respect to the accuracy (t-test) and precision (F-test). No significant differences were found between the calculated and theoretical values of t- and F-tests at 95% confidence level proving similar accuracy and precision in the analysis of the investigated drugs in their dosage forms.

**Table 5:** Determination of H_2_-receptor antagonists-containing dosage forms by the proposed and official methods

Product	Recovery (% ± SD)^a^	F-value^b^	t- value^b^	Official method^c^

Cimetidine^®^ tablets	99.5 ± 0.93	2.11	0.92	98.9 ± 0.64
Famotine^®^ tablets	101.2 ± 0.83	1.44	2.86	99.3 ± 0.69
Servipep^®^ tablets	101.9 ± 1.72	2.15	1.71	100.6 ± 1.15
Peptic^®^ tablets	99.3 ± 1.22	1.51	2.42	97.2 ± 1.50
Famotak^®^ tablets	101.5 ± 1.13	2.68	2.69	99.4 ± 0.69
Antodine^®^ tablets	99.6 ± 1.02	1.95	1.48	98.6 ± 0.73
Gastrodomina^®^ tablets	100.5 ± 0.90	1.60	1.98	99.2 ± 0.71
Antodine^®^ ampoules	102.5 ± 0.79	1.88	1.62	101.5 ± 0.57
Nizatin^®^ capsules	100.3 ± 1.33	1.13	2.69	98.1 ± 1.25
Ranitidine^®^ tablets	98.9 ± 1.15	2.41	0.68	98.4 ± 0.74
Zantac^®^ tablets	99.5 ± 1.25	2.50	2.38	97.3 ± 0.79
Ranitak^®^ tablets	99.7 ± 1.05	2.32	2.78	97.6 ± 0.69
Ranitidol^®^ tablets	98.8 ± 1.18	1.63	1.66	97.2 ± 1.50
Aciloc^®^ tablets	99.5 ± 1.10	1.79	1.42	98.7 ± 0.82
Zantac^®^ ampoules	101.8 ± 1.53	1.49	1.98	100.1 ± 1.25
Ranitidine^®^ ampoules	100.2 ± 1.26	2.97	2.12	98.6 ± 0.73

a Values are the mean of five determinations ± SD;

b Theoretical values for t and F at 95% confidence limit (n=5) were 2.78 and 6.39, respectively;

c Reference (25).

### Stoichiometry and reaction mechanisms

The mole ratio method was used to study the stoichiometry between the investigated drugs and Ce (IV). The results revealed that the Ce (IV):drug ratios were 13:1, 17:1, 15:1, and 19:1 in case of CIM, FAM, NIZ, and RAN, respectively. For accurate interpretation of these high ratios and suggesting the actual reaction mechanism, isolation of the oxidation products was necessary. Experimental work was done for isolation of the oxidation products. The preliminary screening of the reaction mixtures by TLC revealed that many oxidation products were formed, as many spots were seen on the TLC. This was attributed to the presence of many centers that are liable for oxidation in the drug molecule, and support the high values of the molar ratios.

**Figure 3:**
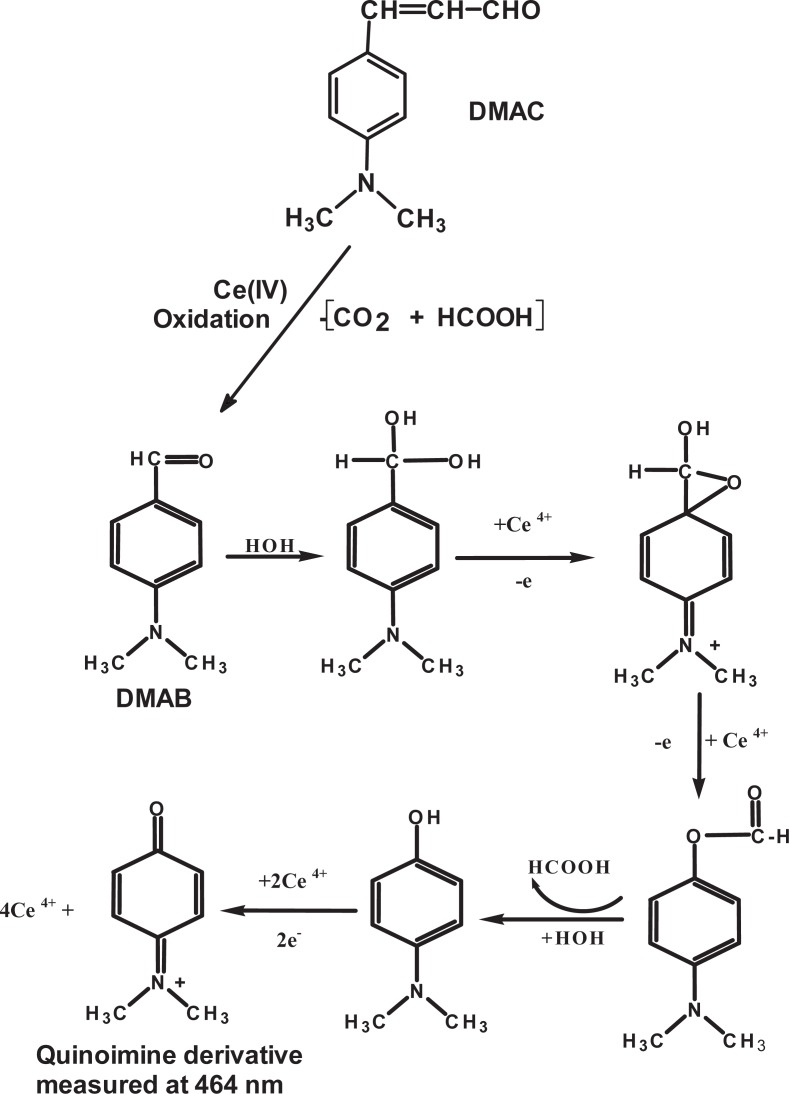
Suggested reaction mechanism of the oxidation of DMAC with Ce (IV).

Using continuous variation method (25), the molar ratio between Ce (IV) and DMAC was found to be 5. Accordingly, the reaction was postulated to proceed according the mechanism given in Figure 3. Treatment of DMAC with Ce (IV) yielded formic acid and p-dimethylaminobenzaldehyde (DMAB). Upon further oxidation of DMAB, p-dimethylaminophenol is produced, as intermediate, and finally the corresponding quinoimine derivative (λ_max_=464 nm) is formed (26). Moreover, the experimental results demonstrating that p-dimethylaminobenzoic acid is not an intermediate, because it failed to produce any color when tested with Ce (IV), confirming that the aldehyde moiety is essential for the color formation. Since p-dimethylaminophenol has no absorption in the visible range (λ_max_=247 nm), the oxidation product of DMAC is postulated to be the quinoimine derivative. This postulation was also supported by report of Lee and Brounridge (27) on the oxidation of cinnamic acid that produces benzaldehyde and formic acid.

## CONCLUSIONS

The results demonstrated the useful use of Ce (IV)/DMAC system in the spectrophotometric analysis of H_2_-RAs. The proposed method was advantageous over other reported visible spectrophotometric methods with respect to its high sensitivity, which permits the determination of a concentration down to 0.12 µg ml^-1^, simplicity of the procedures, and reliability of the results. Furthermore, all the analytical reagents are inexpensive, have excellent shelf life, and are available in any analytical laboratory. The proposed method can be applied in quality control laboratories for the routine analysis of the investigated drugs in raw materials, in pharmaceutical formulations and in the presence of their induced oxidative degradates.
